# Mode of delivery and level of neonatal care in Lombardy: a descriptive analysis according to volume of care

**DOI:** 10.1186/s13052-015-0129-3

**Published:** 2015-03-28

**Authors:** Fabio Parazzini, Sonia Cipriani, Giuseppe Bulfoni, Camilla Bulfoni, Roberto Bellù, Rinaldo Zanini, Fabio Mosca

**Affiliations:** Fondazione IRCCS Cà Granda, Dipartimento Materno-Infantile, Ospedale Maggiore Policlinico, Università degli Studi di Milano, Via Commenda 12, 20122 Milan, Italy; Dipartimento Materno Infantile, Ospedale Buzzi, Università degli Studi di Milano, Via Castelvetro 32, 20154 Milano, Italy; NICU Azienda Ospedaliera della Provinciale di Lecco, Via dell’Eremo 9/11, 23900 Lecco, Italy; Dipartimento di Scienze Cliniche e di Comunità, Universita’ di Milano, Fondazione IRCCS Ca’ Granda Ospedale Maggiore Policlinico, Via Commenda 12, 20122 Milano, Italy; Dipartimento Materno-Infantile Azienda Provinciale Ospedaliera di Lecco, Milan, Italy

**Keywords:** Epidemiology, Volume of care, Neonatal care, Obstetric care

## Abstract

**Background:**

Using data from the Hospital Discharge data-base (SDO) and from the Certificate of Delivery Assistance data-base (CedAP) we analysed mode of delivery and neonatal care in public and private hospitals in Lombardy Region during 2012.

**Methods:**

In Lombardy a standard form is used to register all discharges from public or private hospitals (the SDO data-base which contained information on inpatient activity provided to each patient by any hospital or clinic included in the Regional Health System. Further, information on maternal characteristics and pregnancy outcome are available for all deliveries in CedAP data-base.

We obtained data regarding all deliveries (mother discharge data-base M-SDO)and newborns discharge (N-SDO) and the CedAP data-base over the period January-December 2012 by the Lombardy Health Directorate.

After linkage (using an anonymous key) of the three data-base using anonymized codes we obtained a data-base by the linkage of CedAP and N-SDO records, which includes, after elimination of incorrect codes, information on 90863 neonates and a data-base obtained by the linkage of CedAP and M-SDO records, which includes information on 90868 mother and deliveries. Using these data-base we have analysed mode of delivery and neonatal care in Lombardy according to the volume of care (VoC = number of delivery per year in the care unit).

**Results:**

In 2012, in Lombardy, less than 3% of newborns were born in hospitals reporting less than 500 deliveries/year and less than 30% in hospitals reporting < 1000 deliveries per year. Cesarean section rate was higher in units reporting less than 1000 deliveries/year (28.7% versus 27.5% in hospitals with more than 1000 deliveries/year). In hospitals reporting 500, 500-799, 800-999 deliveries/year the percentage of preterm births with gestational age <33 weeks ranged from 0.1% to 0.2%, but was 3.4% in hospitals reporting 2500 deliveries per year or more. A total of 0.6% of newborns weighing less than 1000 grams and 3.2% of newborns with birth weight between 1000 and 1499 grams was born in hospitals which reported 1000 deliveries or more.

**Conclusions:**

This article provides an overview of delivery and neonatal care in the Lombardy Region with a focus on volume of care.

## Introduction

In the last years a large debate has revolved around obstetric and neonatal care organization, focused on medical aspects and the costs of assistance.

In particular, the effect of volume of obstetric and neonatal care on outcomes and costs has been object of a large debate [1-4].

Regionalization of health care may improve care by directing patients to facilities with the appropriate skill to manage high risk conditions, without increasing costs [2,5].

For example, an US study has suggested that there is benefit to neonatal outcomes when high-risk infants are delivered in hospitals with neonatal intensive care unit (NICU) with high volume of care (VoC) (see Method for definition). This effect, however, differs between states, which may be attributable to different methods of regionalization [1]. Further it has been shown that concentration of high risk deliveries in a smaller number of hospitals has the potential to decrease neonatal mortality without increasing costs [2].

In recent years the availability of administrative data-bases has offered the opportunity of evaluating obstetric and neonatal care on a regional or local basis [6].

Since 1991, a standard form was used to collect information on all discharges from public or private hospitals in Lombardy. These data were available in the Hospital Discharge Data-base (SDO). More recently, the availability of data from all deliveries included in the Certificate of Delivery Assistance data-base (CedAP) has offered the opportunity to integrate the two sources of data to obtain a more detailed information about delivery and neonatal care in the Region.

Using these data we analysed the obstetric and neonatal assistance in public and private hospitals in Lombardy in 2012 according to the VoC of the obstetric/neonatal units. In Lombardy only second level centres (see Methods for definition) are allowed to delivery gestational ages <34 weeks.

## Methods

Since 1991, a standard form was used to register all discharges from public or private hospitals in Lombardy. Data were available in the SDO data-base which contained information on inpatient activity provided to each patient by any hospital or clinic included in the Regional Health System (RHS). The RHS provided reimbursement for hospital admissions to each Regional hospital. On the basis of this information the healthcare providers (both public and private) were reimbursed by the RHS for the services delivered to each patient.

Detailed information regarding maternal characteristics, single and multiple births, pregnancies after assisted reproductive techniques (ART), fetus presentation at delivery, gestational age at birth, induction of labour and mode of delivery, were available for all deliveries in CedAP data-base.

We obtained data regarding all deliveries and neonatal admissions from these two data-base over the period January-December 2012 by the Lombardy Health Directorate.

Due to discrepancies in the data-base period of reference (date of delivery in the CeDAP data-base and date of discharge in the SDO obstetric and neonatal data-base) some numerical differences emerged between the two data-base.

CeDAP data-base included 91655 deliveries and 93244 births (93005 newborns, 239 stillborns).

SDO data-base concerning newborns discharge (N-SDO) included 91830 records about neonates discharged in 2012.

SDO data-base concerning mothers discharge (M-SDO) included 90868 records about women discharged in 2012.

After linkage of the three data-base using anonymized codes we obtained:

- A data-base obtained by the linkage of CedAP and N-SDO records, which includes, after elimination of incorrect codes, information on 90863 neonates;
- A data-base obtained by the linkage of CedAP and M-SDO records, which includes information on 90868 mother and deliveries.

From the SDO data-base, data related to patient hospitalization, diagnosis and discharge were extracted.

From the CedAP data-base, information about maternal characteristics, single and multiple births, pregnancies obtained with ART, fetus presentation at delivery, gestational age at birth and mode of delivery, were extracted.

Information regarding analgesia during labour were obtained from the M-SDO data-base.

Data-base obtained from CeDAP and M-SDO record linkage was used for the analysis of mode of delivery.

Data-base obtained from CeDAP and N-SDO record linkage was used to analyse newborns characteristics.

To verify consistency of data contained in SDO and CedAP records, birth weight values reported in both data-base were compared. In 92.0% of cases no difference was observed. In 6.0% of cases the difference was less than 100 g. Only in 0.7% of cases a difference greater than 500 g was observed.

According to the regional law we defined “Level I” care unit those reporting <1000 deliveries/year; “Level II” those reporting 1000 or more deliveries, with or without neonatal intensive care.

All the analyses were carried out using SAS/STAT, version 9.2 software (SAS Institute Inc, Cary, NC, USA).

## Results

Table 1 showed the distribution of 74 obstetric/neonatal units of the RHS according to the VoC.

**Table 1:** **Distribution of obstetric/neonatal units according to volume of care (number of deliveries/year)**

**Volume of care**	**Hospitals (N = 74)**		**Deliveries N = 91601**	
	**N**	**%**	**N**	**%**
<500	10	13.5	2676	2.9
500-799	20	27.0	12608	13.8
800-999	11	14.9	9965	10.9
1000-2499	25	33.8	37860	41.3
≥2500	8	10.8	28492	31.1

40.5% of the obstetric/neonatal care units reported less than 800 deliveries: in these centres only 16.7% of newborns were delivered.

A total of 28492 deliveries (31.1%) occurred in 8 centres reporting more than 2500 deliveries.

The mean maternal age at birth was 32.4 years (range 14–54). In particular, it was 31.5 years for the first, 32.7 years for the second, 33.8 years for the third, 35.0 years for the fourth born or more. In 9.0% of births maternal age was ≥ 40 years.

Mother was foreigner in 26351 deliveries (28.8%). ART was reported in 2264 (2.5%) deliveries.

A total of 1555 multiple deliveries occurred (3156 newborns (3.4%)). Of these, 3044 (3.3%) were twin deliveries, 96 (0.1%) triplets and 4 (0.004%) quadruplets.

Finally, 4.5% of births were recorded with breech or other abnormal presentations.

During the 2012 caesarean section rate was 27.8%. Vacuum was reported in 6.2% of deliveries.

Eleven point six percent of births were recorded in women who reported one or more previous caesarean sections (7.9% one, 1.2% two and 0.2% 3 or more previous caesarean section). Vaginal birth after caesarean section occurred in 18.1% of cases.

Cesarean section rate was higher in units reporting less than 1000 deliveries per year (Table 2).

**Table 2:** **Mode of delivery according to the volume of care (number of deliveries/year in the care unit)**

**Volume of care**	**Deliveries**	**Vaginal delivery**							**Cesarean section**						**Induced labour**		**CS after induction**		**Women with previous CS**		**Vaginal after CS (VBAC)**	
		**Total vaginal delivery**		**Normal vaginal delivery**		**Vacuum**		**Forceps**	**Total CS**		**Elective CS**		**Emergency CS**									
	**N**	**N**	%*	**N**	%*	**N**	%*	**N**	**N**	**%**	**N**	%*	**N**	**%**	**N**	%*	**N**	%**	**N**	%*	**N.**	%***
<500	2676	1749	65.4	1622	60.6	127	4.7	1	926	34.6	604	65.2	322	34.8	628	23.5	124	19.7	432	16.1	28	6.5
500-799	12608	8746	69.4	8268	65.6	478	3.8	1	3861	30.6	1987	51.5	1874	48.5	2400	19.0	617	25.7	1600	12.7	178	11.1
800-999	9965	7500	75.3	7048	70.7	452	4.5	0	2465	24.7	1465	59.4	1000	40.6	1757	17.6	347	19.7	1121	11.2	189	16.9
1000-2499	37860	27901	73.7	26228	69.3	1673	4.4	0	9959	26.3	4456	44.7	5503	55.3	7023	20.3	1377	19.6	4166	11.0	761	18.3
≥2500	28492	20236	71.0	18891	66.3	1345	4.7	0	8256	28.9	3681	44.6	4575	55.4	5642	19.8	1087	19.3	3298	11.6	762	23.1
Total	91601	66132	72.2	62057	67.7	4075	4.4	2	25467	27.8	12193	47.9	13274	52.1	17450	19.8	3552	20.4	10617	11.6	1918	18.1

*Row percentage of total number of deliveries in the volume of care category.

**Row percentage of total number of deliveries in the volume of care category excluding missing data on induction information (n = 3286).

***Row percentage of deliveries of women with previous CS.

CS = Cesarean section.

Labours were induced in 17450 cases (19.8%). Among those, a caesarean section was reported in the 20.4% of cases. No significant differences were observed in the frequency of induction and caesarean section after induction according to volume of care.

Analgesia in labour was performed in 21.1% of vaginal deliveries. A percentage higher than 25% was observed in 22 centres with no particular differences between volume of care (data not shown).

Table 3 shows the distribution of births by gestational week at birth and volume of care.

**Table 3:** **Gestational week at delivery according to the volume of the obstetric/neonatal unit**

**N. of deliveries per year**	**Gestational week at delivery**										
	**<22**		**22-29**		**30-33**		**34-36**		**≥37**		**Total**
	**N**	%*	**N**	%*	**N**	%*	**N**	%*	**N**	%*	**N**
<500	0	--	3	0.1	6	0.2	98	3.7	2576	96.0	2683
500-799	0	--	3	0.0	18	0.1	527	4.2	12112	95.7	12660
800-999	0	--	13	0.1	21	0.2	463	4.6	9536	95.0	10033
1000-2499	13	0.0	226	0.6	614	1.6	2301	6.0	35268	91.8	38422
≥2500	13	0.0	301	1.0	665	2.3	1900	6.5	26274	90.1	29153
Totale	26	0.0	546	0.6	1324	1.4	5289	5.7	85766	92.3	92951

*Row percentage.

The percentage of births ranged from 0.1% to 0.2% in units reporting less than 500, 500–799 and 800–999 deliveries, but was 3.3% in units reporting 2500 deliveries or more.

A total of 291 newborns (0.3%) weighing less than 1000 grams, and 584 (0.6%) between 1000 and 1499 grams was reported. Birth weight greater than 4000 grams was recorded in 4889 cases (5.4%).

Table 4 showed the distribution of births by birth weight according to volume of care: 0.6% of newborns weighing less than 1000 grams and 3.2% of those with birth weight ranging between 1000 and 1499 grams was born in hospitals reporting 1000 births or more.

**Table 4:** **Birth at weight according to the volume of care (number of deliveries per year in the care unit)**

**N. of deliveries per year**	**Weight at birth (g)**											
	**<1000**		**1000-1499**		**1500-1999**		**2000-2499**		**2500-3999**		**≥4000**	
	**No.**	**%**	**No.**	**%**	**No.**	**%**	**No.**	**%**	**No.**	**%**	**No.**	**%**
<500	0	0.0	7	1.2	12	1.0	89	2.0	2208	2.8	110	2.2
500-799	1	0.3	3	0.5	38	3.1	468	10.6	11213	14.1	737	15.1
800-999	1	0.3	9	1.5	55	4.5	368	8.8	8806	11.1	577	11.8
1000-2499	120	41.2	252	43.2	545	45.0	1878	42.2	32832	41.3	2060	42.1
≥2500	169	58.1	313	53.6	560	46.3	1603	36.4	24424	30.7	1405	28.7
Totale	291	0.3	584	0.6	1210	1.3	4406	4.8	79483	87.5	4889	5.4

A total of 160 (0.2%) newborns reported an Apgar’s score <5 at 5 minutes. Of those 7 were observed in units reporting less than 500 deliveries.

Table 5 showed the distribution of births according to level of care.

**Table 5:** **Distribution of births by care unit level**

**Level I**		**Level II**		**Level II NICU**	
**N**	**%**	**N**	**%**	**N**	**%**
25029	27.3	18981	20.7	47820	52.1

NICU = Neonatal intensive care unit.

A total of 0.8% of newborns were admitted to another hospital after birth.

A respiratory distress syndrome (RDS) was reported in 1123 births (1.2%) (Table 6).

**Table 6:** **Frequency of Respiratory Distress Syndrome (RDS) according to birth weight**

			**Weight at birth (g )**											
	**Total deliveries**		**<1000**		**1000-1499**		**1500-1999**		**2000-2499**		**2500-3999**		**≥4000**	
	**N**	%*	**N**	%*	**N**	%*	**N**	%*	**N**	%*	**N**	%*	**N**	%*
Births with RDS	1123	1.2	281	96.6	326	55.8	221	18.3	128	2.9	160	0.2	7	0.1

*Percentage of total deliveries in the birth weight category.

## Discussion

The results of this analysis provided a global view of delivery and neonatal care in Lombardy in 2012 in units with low and high level of care.

Our data cannot be considered totally representative of all deliveries. In fact some deliveries and infants have not been included due to lack of link between SDO and CedAP data. This is a limitation of this analysis: excluded infants could be systematically different from the total population.

With regard to quality of diagnosis, for administrative reasons, all medical records are reviewed and diagnosis are confirmed by local medical officers.

Among the strengths, we also have to consider the population-based design and the opportunity to analyse temporal trends using similar methods of data collection.

In Lombardy currently less than 3% of newborns are delivered in units reporting less than 500 deliveries/year and less than 30% in units reporting less than 1000 deliveries/year.

These findings were lower than national ones. In Italy, in 2010, the 7.1% of babies were born in centres reporting 500 or less deliveries/year and 32.1% in centres reporting <1000 deliveries/year [7].

In 2012 in Lombardy 2.5% deliveries occurred in women who underwent ART. This frequency is steadily increasing [8]. These pregnancies were characterized by a high frequency of low birth weight (birth weight ≤1500 grams): 156/1013 (15.4%) of births with a birth weight less than 1500 grams were observed in women who underwent ART (data not shown).

During 2012 breech or abnormal fetus presentation at delivery were detected in 4.5% of newborn. This frequency was 3.5% during 2002. This increase could be explained by higher maternal age and higher frequency of pregnancies after ART both recognised risk factors for breech or abnormal presentation [9,10].

A goal of present study was to describe mode of delivery and burden of neonatal care, using gestational age and birth weight as indicator of high care level.

Caesarean section rate in 2012 in the Lombardy Region was 27.8%. This frequency was decreasing in comparison with previous years [11]. This recent trend is partially due to the increasing frequency of vaginal deliveries after caesarean section. In Lombardy, the percentage was 18.1% in 2012, but 7.3% in 2002 and 6.9% in 2008. In 15 centres the frequency of vaginal delivery was 20% or more in women with previous caesarean section (data not shown).

Health care units offering more delivery services were assumed to provide better quality of care and one of the indicators of good quality of care is a low frequency of CS.

It has been suggested that, after taking into account the level of mothers’ risk of CS, significant differences in risk-adjusted CS rates in the below-average- and medium-risk groups are present between low- and high-volume hospitals [12]. In our analysis the CS rates were higher in small units. This finding may be explained, at least in part, by the lack of 24 hours anestesiological facilities in these units. Similar findings also emerged in an analysis referred to 2009 [13].

Regionalization may prevent high risk deliveries in low volume units leaving only low risk women to delivery safely in these units. In Australia, lower hospital volume was not associated with adverse outcomes for low risk women [14].

We analysed if regionalization directed patients to facilities with the appropriate capabilities to manage low birth weight newborns.

Newborns with low gestational age were rarely observed in units with low volume of care (<1000 deliveries/year). In fact, only 57 newborns with a gestational age less than 34 weeks of gestation were born in 2012 in these units and only 0.8% of all newborns were transferred to another hospital after birth.

Level I neonatal care units delivered almost exclusively low risk pregnancies at term.

Along this line, respiratory distress syndrome (RDS) has been reported in about 1% of the N-SDO data-base records. In newborns with birth weight less than 2000 grams 828 (39.7%) presented RDS.

In conclusion, the present article provided an overview about delivery and neonatal care in Lombardy Region. The analysis of administrative regional [8,11,15,16] and national [7] data-base were useful to improve care. A continuous monitoring of regional and national practice and the improvement of the quality of data collection, will be in the next years a powerful analytical tool for the identification of care priorities.

## References

[1] Lorch SA, Baiocchi M, Ahlberg CE, Small DS (2012). The differential impact of delivery hospital on the outcomes of premature infants. Pediatrics.

[2] Phibbs CS, Bronstein JM, Buxton E, Phibbs RH (1996). The effects of patient volume and level of care at the hospital of birth on neonatal mortality. JAMA.

[3] Phibbs CS, Baker LC, Caughey AB, Danielsen B, Schmitt SK, Phibbs RH (2007). Level and volume of neonatal intensive care and mortality in very-low-birth-weight infants. N Engl J Med.

[4] Watson SI, Arulampalam W, Petrou S, Marlow N, Morgan AS, Draper ES (2014). The effects of designation and volume of neonatal care on mortality and morbidity outcomes of very preterm infants in England: retrospective population-based cohort study. BMJ Open.

[5] Lorch SA, Myers S, Carr B (2010). The regionalization of pediatric health care. Pediatrics.

[6] Knight HE, Gurol-Urganci I, Mahmood TA, Templeton A, Richmond D, van der Meulen JH (2013). Evaluating maternity care using national administrative health datasets: how are statistics affected by the quality of data on method of delivery?. BMC Health Serv Res.

[7] Basili F, Cocchi M, Di Rosa A, Tamburini C. Certificato di assistenza al parto. Analisi dell’evento nascita –Anno 2010. http://www.salute.gov.it/imgs/c_17_pubblicazioni_2024_allegato.pdf (last access 23 sept 2014). 2010.

[8] Parazzini F, Cipriani S, Ricci E, Bulfoni G, Natale N, Frigerio L (2011). I ricoveri ostetrici nella Regione Lombardia nel 2008. Parte II. It J Gynecol Obstet.

[9] Romundstad LB, Romundstad PR, Sunde A, von During V, Skjaerven R, Vatten LJ (2009). Assisted fertilization and breech delivery: risks and obstetric management. Hum Reprod.

[10] Hofmeyr GJ, Hannah ME. Planned Caesarean section for term breech delivery. Cochrane Database Syst Rev [1361-6137]: 2001; 1:CD000166. 10.1002/14651858.CD000166 11279680

[11] Parazzini F, Bulfoni G, Cipriani S, E R (2010). I ricoveri ostetrici negli ospedali della Lombardia nel 2008. It J Gynaecol Obstet.

[12] Lee KS, Kwak JM (2014). Effect of patient risk on the volume-outcome relationship in obstetric delivery services. Health Policy..

[13] Zanini A, Lissoni D, Andreotti C, Spreafico C, Miglietta M, Pirovano C (2013). Robson class I Cesarean section in Lombardia: what is happening?. It J Gynecol Obstet.

[14] Tracy SK, Sullivan E, Dahlen H, Black D, Wang YA, Tracy MB (2006). Does size matter? A population-based study of birth in lower volume maternity hospitals for low risk women. BJOG.

[15] Parazzini F, Ricci E, Cipriani S, Chiaffarino F, Bortolus R, Chiantera V (2013). Temporal trends and determinants of peripartum hysterectomy in Lombardy, Northern Italy, 1996–2010. Arch Gynecol Obstet.

[16] Parazzini F, Ricci E, Cipriani S, Motta T, Chiaffarino F, Malvezzi M (2013). Temporal trends in adolescent pregnancies in Lombardy, Italy: 1996–2010. Eur J Contracept Reprod Health Care.

